# A Review of β-Ga_2_O_3_ Power Diodes

**DOI:** 10.3390/ma17081870

**Published:** 2024-04-18

**Authors:** Yongjie He, Feiyang Zhao, Bin Huang, Tianyi Zhang, Hao Zhu

**Affiliations:** 1 School of Microelectronics, Fudan University, Shanghai 200433, China; 22212020007@m.fudan.edu.cn (Y.H.); 23212020203@m.fudan.edu.cn (F.Z.); 23212020081@m.fudan.edu.cn (B.H.); 23212020196@m.fudan.edu.cn (T.Z.); 2 National Integrated Circuit Innovation Center, Shanghai 201203, China

**Keywords:** β-Ga_2_O_3_, Schottky barrier diodes (SBDs), heterojunction diodes (HJDs), power devices

## Abstract

As the most stable phase of gallium oxide, β-Ga_2_O_3_ can enable high-quality, large-size, low-cost, and controllably doped wafers by the melt method. It also features a bandgap of 4.7–4.9 eV, a critical electric field strength of 8 MV/cm, and a Baliga’s figure of merit (BFOM) of up to 3444, which is 10 and 4 times higher than that of SiC and GaN, respectively, showing great potential for application in power devices. However, the lack of effective p-type Ga_2_O_3_ limits the development of bipolar devices. Most research has focused on unipolar devices, with breakthroughs in recent years. This review mainly summarizes the research progress fora different structures of β-Ga_2_O_3_ power diodes and gives a brief introduction to their thermal management and circuit applications.

## 1. Introduction

As technology advances, there is an increasing need for electronic devices capable of a high frequency, high voltage, and high power in various applications, such as AI, 5G communication, electric vehicles, rail transportation, new energy power generation, and power transmission [1,2,3,4]. Si, being the most mature semiconductor material, is widely used in electronic manufacturing, but its narrow bandgap (1.12 eV) significantly limits its application in high-voltage fields. Si-based power devices are gradually reaching their performance limits but still cannot meet the growing demands for development [5,6,7]. Consequently, wide-bandgap semiconductor materials, represented by SiC (3.3 eV) and GaN (3.4 eV), have progressively entered the spotlight. Owing to their larger bandgap, the device’s maximum breakdown field strength can be improved, and their higher electron mobility and saturation velocity result in lower conduction losses and switching losses. Hence, wide-bandgap power semiconductor devices have witnessed a rapid development, serving to address the shortcomings of Si-based power devices in the field of high-power electronics [8,9]. However, the production cost of SiC and GaN is significantly higher than that of Si wafers, as these materials require epitaxial growth methods to obtain high-quality crystals, which severely limits their large-scale application [10,11,12].

As a quintessential exemplar of ultra-wide-bandgap semiconductors, Ga_2_O_3_ manifests in five polymorphic phase structures, encompassing the α, β, γ, δ, and ε phases. Among them, the β phase is endowed with thermodynamic stability. Utilizing melt growth methodologies, it is feasible to fabricate high-quality, large-scale, cost-effective, and doping-controllable β-Ga_2_O_3_ wafers. Furthermore, several pivotal electrical properties of β-Ga_2_O_3_ exhibit superiority over those inherent to SiC and GaN [13,14,15,16,17,18,19]. Currently, 6-inch β-Ga_2_O_3_ wafers have been successfully fabricated using the edge-defined film-fed growth (EFG) technique. The cost of β-Ga_2_O_3_ wafers produced via the Czochralski (CZ) method amounts to only one-third of that for SiC wafers of the same size. Notably, the EFG method proves even more cost-effective, being two times less expensive than the CZ process, thus rendering the overall cost of β-Ga_2_O_3_ substantially lower than that of SiC [20,21]. Moreover, during the production of β-Ga_2_O_3_ wafers, the cost and attrition rate of Ir crucibles constitute a significant proportion of expenditure. In April 2022, C&A Corporation (Sendai, Japan) succeeded in growing 2-inch β-Ga_2_O_3_ single crystals using a Cu crucible CZ method, potentially reducing costs to one-hundredth of those associated with the EFG method, laying the foundation for the mass application of β-Ga_2_O_3_ devices [22]. Electrically, β-Ga_2_O_3_ possesses a wider bandgap of 4.7–4.9 eV and a critical electric field strength of 8 MV/cm, which is 27 times that of Si, 3 times that of SiC, and 2 times that of GaN. The Baliga’s figure of merit (BFOM), a crucial parameter for evaluating the suitability of materials for power device fabrication, reaches 3444 for β-Ga_2_O_3_, which is 10 times that of SiC and 4 times that of GaN; this indicates that β-Ga_2_O_3_ possesses greater potential for application in power devices [23,24,25,26].

Although β-Ga_2_O_3_ offers the advantages of low cost and high performance, similar to other wide-bandgap semiconductor materials, it is challenging to obtain p-type Ga_2_O_3_ with a high conductivity [27,28,29,30]. Calculations suggest the absence of shallow acceptors in Ga_2_O_3_, and holes exhibit self-trapping effects within the material [31,32]. Consequently, current research is primarily focused on unipolar Ga_2_O_3_ power electronic devices, including field-effect transistors (FETs) and Schottky barrier diodes (SBDs) [33,34,35]. Compared to traditional p-n junction diodes, SBDs exhibit lower turn-on voltages and faster recovery times, making them commonly employed in low-power and high-speed switching applications. However, the breakdown voltage (BV) of conventional Si-based SBDs is generally low, and the introduction of wide-bandgap materials can effectively elevate their breakdown limits. Since Sasaki et al. [36] first reported on β-Ga_2_O_3_ SBDs in 2012, β-Ga_2_O_3_ SBD devices have been developing towards a higher BV and lower on-resistance (R_on_) through improvements in epitaxial layer quality, device structure enhancements, and optimized manufacturing processes.

In recent years, there has been a sharp increase in research publications on Ga_2_O_3_ SBDs, leading to significant breakthroughs in device performance. Lateral β-Ga_2_O_3_ SBDs have achieved a BV exceeding 10 kV [37,38], and vertical NiO/β-Ga_2_O_3_ Schottky heterojunction diodes devices have reached a power figure of merit (PFOM = BV^2^/R_on_) of 13.2 GW/cm^2^ [39]. Significant progress has also been made in commercial applications; in December 2021, Novel Crystal Technology Inc. (Saitama, Japan) announced the release of an ampere-level 1200 V β-Ga_2_O_3_ trench SBD device [40]. Therefore, it is essential to compile a summary of the current research progress on β-Ga_2_O_3_ power SBD devices, as it may provide meaningful guidance for future developments in the field.

This article provides a comprehensive review of the research advancements in variously structured β-Ga_2_O_3_ power SBDs up to the year 2024. Section 2 elucidates the material properties of β-Ga_2_O_3_, substrate preparation, and epitaxial processes; Section 3 discusses the metal/β-Ga_2_O_3_ contacts, including ohmic and Schottky contacts; Section 4 presents the progress in research on differently structured β-Ga_2_O_3_ power diodes, which encompasses vertical structure SBDs, heterojunction structure diodes, and lateral structure diodes; Section 5 offers a brief overview of the surge current robustness and thermal management of β-Ga_2_O_3_ SBD devices; Section 6 summarizes the circuit applications and reliability of β-Ga_2_O_3_ SBDs; and Section 7 concludes with a summary and prospects.

## 2. β-Ga_2_O_3_ Materials

**Figure 1:**
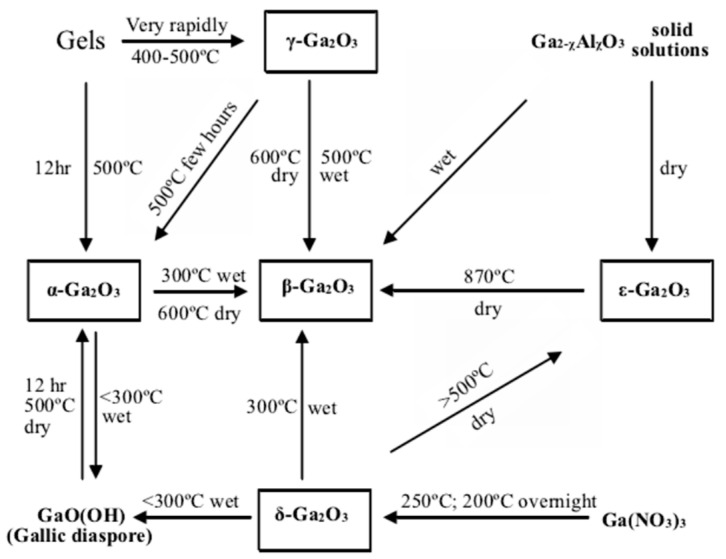
Transformation relationships between different crystal phases of β-Ga_2_O_3_ [13].

Ga_2_O_3_ material can exist in five crystal phases (α, β, γ, δ, and ε), among which the monoclinic β-Ga_2_O_3_ phase prevails instability. The remaining four phases are metastable and can be converted to β-Ga_2_O_3_ under certain conditions, as depicted in Figure 1 [13,14]. Notably, β-Ga_2_O_3_ exhibits commendable thermal and physicochemical stability, making it the most extensively studied phase to date. The α-Ga_2_O_3_ phase possesses a corundum structure and is typically grown heteroepitaxial on sapphire substrates [41,42,43,44], making it the second-most researched phase. The FLOSFIA company (Kyoto, Japan) predominantly employs Mist-epitaxy to prepare α-Ga_2_O_3_ wafers, and in March 2023, they unveiled an amperage-class 1700 V α-Ga_2_O_3_ trench SBD device [45,46,47,48,49]. The cubic spinel structure of γ-Ga_2_O_3_ is principally utilized as a photocatalyst for hydrogen production via water splitting [50,51,52,53]. Current research on the cubic perovskite structure of δ-Ga_2_O_3_ is comparatively limited. The hexagonal structure of ε-Ga_2_O_3_, which exhibits spontaneous polarization, can form a high-density two-dimensional electron gas at heterojunction interfaces and holds the potential for fabricating high-electron-mobility transistors [54,55]. Furthermore, there exists an orthorhombic κ-Ga_2_O_3_ with a very similar crystal-structure ε-Ga_2_O_3_, and they are usually considered as one structure [56,57]. Recently, orthorhombic κ-Ga_2_O_3_ has been proven to be stable up to 800–1000 °C, and this makes it suitable for the fabrication of reliable devices such as detectors for UV and X-rays [58,59,60,61].

**Figure 2:**
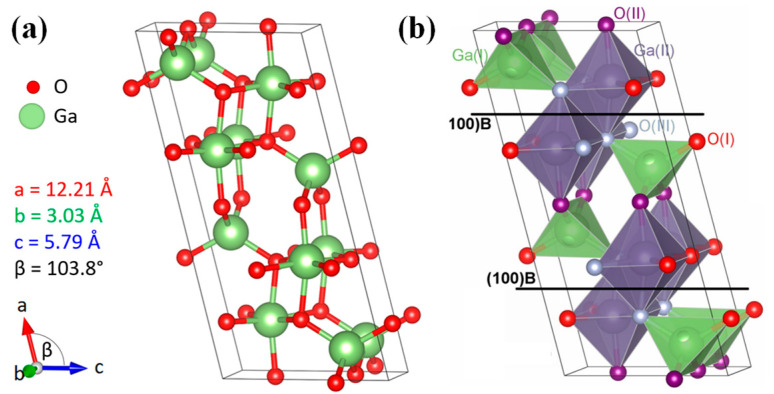
Schematic representation of β-Ga2O3 (**a**) crystal structure and (**b**) unit cell [62].

Monoclinic-phase β-Ga_2_O_3_ belongs to the C2/m space group with a densely stacked anion structure. Its crystal structure and unit cell schematic are shown in Figure 2 [19,62]. The unit cell is comprised of two GaO_4_ tetrahedra and two GaO_6_ octahedra, containing two different Ga atomic positions, as well as three distinct O atomic sites. The lattice constants of β-Ga_2_O_3_ are as follows: a = 12.21 Å, b = 3.03 Å, c = 5.79 Å, with α = γ = 90° and β = 103.8°. The length of the a-axis is four times that of the b-axis, and the length of the c-axis is 1.9 times that of the b-axis, which facilitates cleavage along the (100) and (001) directions. Utilizing these crystallographic properties of β-Ga_2_O_3_, it is feasible to perform mechanical exfoliation to create quasi-two-dimensional thin-layer materials. This attribute endows β-Ga_2_O_3_ with significant potential in the domain of two-dimensional materials and devices [63,64,65,66].

The growth techniques for β-Ga_2_O_3_ single-crystal substrates predominantly encompass the Czochralski method (CZ) [15,18,67], optical floating zone method (OFZ) [68,69,70], vertical Bridgman method (VB) [71,72,73], and edge-defined film-fed growth method (EFG) [17,74,75,76,77], among which the EFG is the most mature, offering the potential for mass production and cost efficiency. In 2018, Japan’s Novel Crystal Technology Inc. (Saitama, Japan) pioneered the fabrication of 6-inch β-Ga_2_O_3_ single-crystal substrates using the EFG method. The company has also industrialized the production of 4-inch β-Ga_2_O_3_ single-crystal substrates, currently holding an international leading position, thereby laying the foundation for the large-scale commercialization of gallium oxide devices.

Advancements in substrate materials have also facilitated the development of β-Ga_2_O_3_ homoepitaxial film technologies. The epitaxial techniques widely employed today include molecular beam epitaxy (MBE) [78,79,80], halide vapor phase epitaxy (HVPE) [81,82,83,84], metal–organic chemical vapor deposition (MOCVD) [85,86,87], pulsed laser deposition (PLD) [88,89,90], low-pressure chemical vapor deposition (LPCVD) [91,92,93], and mist chemical vapor deposition (Mist-CVD) [43,94,95]. Among these, HVPE has been distinguished for achieving high growth rates of approximately 28 µm/h for epitaxial films. It also allows for precise doping concentration control within the range of 10^15^ to 10^19^ cm^−3^. At present, the majority of the commercially produced β-Ga_2_O_3_ single-crystal epitaxial layers are fabricated utilizing HVPE technology.

As compared to the already implemented SiC and GaN, the β-Ga_2_O_3_ material exhibits superior properties, as shown in Table 1 [19,96]. The bandgap of β-Ga_2_O_3_ ranges from 4.7 to 4.9 eV, with its ultraviolet absorption edge situated between 260 nm and 280 nm, precisely aligning with the solar-blind UV spectrum of 200 nm to 280 nm. This intrinsic property renders β-Ga_2_O_3_ a rare and ideally suited material for solar-blind UV detection. Consequently, Ga_2_O_3_-based solar-blind UV photodetectors have been extensively investigated [97,98,99,100]. Furthermore, β-Ga_2_O_3_ features a maximum critical electric field strength of up to 8 MV/cm and a BFOM of 3444. This implies that β-Ga_2_O_3_ devices have the theoretical capability to withstand a higher BV and operate under higher power conditions, making them more suitable for power device applications. Although diamond has superior properties in comparison, it is still far from being commercially viable, due to the limit of its large size and high-quality single crystal preparation, as well as its extremely high production and processing costs [101,102]. Interestingly, diamond is capable of effective p-type doping but lacks n-type doping and possesses a very high thermal conductivity. Thus, the complementary formation of a heterojunction diode with β-Ga_2_O_3_ can give full play to the advantages of both. A diamond/β-Ga_2_O_3_ pn heterojunction diode with on/off ratios of greater than 10^8^ at ±10 V and leakage currents of less than 10^−12^ A has already been obtained by a direct bonding method [103,104].

At present, Ga_2_O_3_ materials still lack effective p-type doping, and research applications are basically n-Ga_2_O_3_, typically doping with elements like Si and Sn within a range of 10^15^ to 10^19^ cm^−3^ in concentration. In recent years, researchers have begun to address the shortfall of p-type Ga_2_O_3_ by using p-type NiO to form a p-n heterojunction with Ga_2_O_3_, presenting a novel approach for the advancement of Ga_2_O_3_ devices [39,105]. Besides, the primary challenges associated with β-Ga_2_O_3_ materials are their relatively low electron mobility and thermal conductivity. β-Ga_2_O_3_ exhibits an electron mobility of 300 cm^2^/V·s compared to GaN, rendering it less ideal for the fabrication of high-frequency devices. However, its saturation electron velocity of 2 × 10^7^ cm/s offsets this disadvantage [106,107]. In response to potential heat dissipation issues due to the limited thermal conductivity of Ga_2_O_3_ materials, numerous studies have proposed solutions, such as hetero-bonding, substrate thinning, and junction-side cooling, which will be elaborated in Section 5. Overall, Ga_2_O_3_ devices possess considerable practical application value and hold broad prospects for utilization.

## 3. Metal/β-Ga_2_O_3_ Contact

The fundamental structure of β-Ga_2_O_3_ SBDs is composed of β-Ga_2_O_3_ and an anodic Schottky contact, as well as a cathodic ohmic contact. The contact between metal and β-Ga_2_O_3_ is the principal determinant of interfacial electron transport and significantly impacts the performance of the device.

### 3.1. Schottky Contact

**Figure 3:**
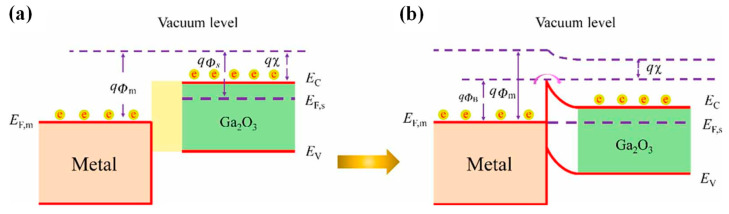
Schematic energy band diagrams of a metal/β-Ga_2_O_3_ Schottky contact: (**a**) before contact and (**b**) after contact [108].

When the metal and semiconductor are in contact, the diffusion of charge carriers equalizes the Fermi levels on both sides of the interface, reaching an equilibrium state. Consequently, after contact, the energy bands in the semiconductor become bent due to the built-in electric field, thereby forming an electron potential barrier at the interface, known as the Schottky barrier, as illustrated in Figure 3 [108]. The Mott model posits that the height of the Schottky barrier is solely related to the metal work function and the electron affinity of the semiconductor, and the Schottky barrier height (SBH) is equal to the difference between the two [109]. Experimentally, the SBH is commonly determined through current–voltage (I-V) or capacitance–voltage (C-V) measurements. Currently, there are many metals that are capable of forming Schottky contacts with β-Ga_2_O_3_, including Pt, Ir, Pd, Au, Ni, Co, Ru, Cu, Mo, W, Cr, Ti, Ag, etc.; graphite also forms Schottky contacts with β-Ga_2_O_3_. Among these, Ni is widely used, due to its low cost and ability to form excellent Schottky contacts with β-Ga_2_O_3_ [110,111,112,113]. 

However, experimental results indicate that the SBH of β-Ga_2_O_3_ weakly depends on the metal work function and is more influenced by interface states, as well as interface structures and interactions [114]. The Bardeen model suggests that electron transfer from the semiconductor to the metal is mediated by interface states on the contact surface, assuming a continuous distribution of surface states defined by a neutral energy level Φ_0_, with the Fermi level position determined by the location of the surface states within the bandgap, which is the Fermi level pinning effect [115]. Therefore, the surface states of β-Ga_2_O_3_ also significantly affect device performance; experiments have found that treatments such as oxygen plasma and annealing, as well as chemical solution cleaning, can effectively reduce the density of surface states on β-Ga_2_O_3_, enhancing device performance, while etching and plasma bombardment may increase surface state density, leading to poorer device performance [116,117,118,119,120,121]. 

In practical applications, due to the amount of heat generated by high power, power devices often operate in high-temperature environments, and many studies have investigated the high-temperature performance of different Schottky metal/β-Ga_2_O_3_ SBDs [122,123,124]. Furthermore, given that metal oxides have a better high-temperature stability and higher SBH compared to metals, β-Ga_2_O_3_ SBDs utilizing metal oxides as Schottky electrodes have also been the subject of extensive research [125,126,127]. Hou et al. [128] reported that IrO_x_/β-Ga_2_O_3_ Schottky contacts achieved a leakage current of only 2.3 × 10^−9^ A/cm^2^ (@-3 V) at a high temperature of 350 °C and just 7.5 × 10^−8^ A/cm^2^ at −100 V, with the rectification ratio exceeding 10^10^ at all temperatures. Dela Cruz et al. [129] showed the outstanding high-temperature performance of Pt_x_Ir_(1−x)_O_y_ on ($\bar{2}$01) β-Ga_2_O_3_, with a rectification ratio of 10^9^ (±3 V) at 300 °C and 10^6^ at 500 °C, demonstrating the application potential of metal oxide Schottky contacts in high-temperature devices.

### 3.2. Ohmic Contact

Typically, an excellent ohmic contact exhibits a low or even no SBH, manifesting a linear I-V curve that minimizes thermal effects caused by contact resistance. This is particularly crucial for β-Ga_2_O_3_, which has a low thermal conductivity. Hence, superior β-Ga_2_O_3_ ohmic contacts are a prerequisite for achieving high-performance devices. Currently, almost all Ga_2_O_3_ devices use Ti as the ohmic contact and deposit Au as a protective layer to avoid oxidation. The ohmic contacts can be effectively formed by heavy doping, plasma treatment, and post-annealing treatment [110,111,112,113]. In 2012, Higashiwaki et al. [130] first reported a field-effect transistor based on β-Ga_2_O_3_, utilizing Ti/Au ohmic contacts, and highlighted the necessity of Reactive Ion Etching (RIE) processing for establishing ohmic contacts. Subsequently, the ohmic contact of Ti on β-Ga_2_O_3_ was improved in β-Ga_2_O_3_ MOSFETs devices through annealing at 470 °C for 1 min [131]. Bhattacharyya et al. [132] reported a record low contact resistivity of 80 mΩ·mm for Ti metal on heavily doped (∼1.8 × 10^20^ cm^−3^) β-Ga_2_O_3_, with a specific contact resistivity of 8.3 × 10^−7^ Ω·cm^2^. Currently, the formation of high-quality Ti/β-Ga_2_O_3_ ohmic contacts is commonly achieved using post-annealing treatment.

## 4. β-Ga_2_O_3_ Power Diodes

In the last decade, β-Ga_2_O_3_ power diodes have been extensively studied due to their exceptional electrical properties. To enhance device performance, researchers have proposed new device structures, which can be categorized into three main groups based on their structure type: vertical-structure SBDs, vertical heterojunction-structure diodes, and lateral-structure diodes.

### 4.1. Vertical-Structure SBDs

SBDs are unipolar devices that benefit from significantly reduced switching losses due to the absence of minority carrier storage effects. Initial β-Ga_2_O_3_ SBDs exhibited excellent rectifying behavior, but the device BV was low due to the electric field concentration effect at the electrode edges. To address this, researchers have employed various structures to alleviate electric field concentration, thereby enhancing the BV and overall device performance. These structures include field plates (FPs), edge termination (ET), mesa termination (MT), and trench structure. Beyond the BV, device performance parameters also encompass ideality factors (n), on-state voltage (V_on_), on-resistance (R_on_), forward current (I_F_), and reverse leakage current. Amongst these, the BV and R_on_, as well as their derived PFOM, are particularly crucial, reflecting the device’s potential in power circuit applications. This section summarizes the development of devices with different vertical structures of β-Ga_2_O_3_ SBDs.

#### 4.1.1. Simple Structure

**Figure 4:**
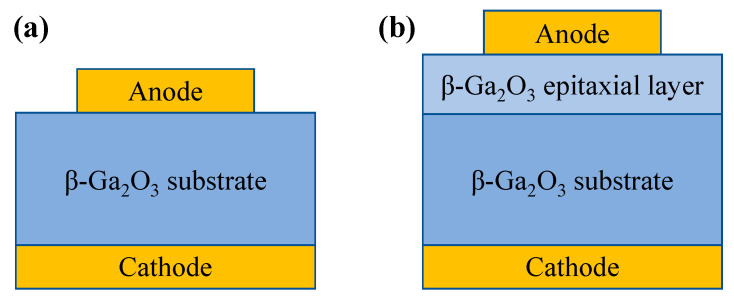
Device schematic of vertical β-Ga_2_O_3_ SBDs on (**a**) β-Ga_2_O_3_ substrate and (**b**) with β-Ga_2_O_3_ epi-layer on β-Ga_2_O_3_ substrate.

Early devices were fabricated on single-crystal substrates and exhibited good rectifying properties. However, their breakdown performance was significantly inferior to bipolar devices, as they relied only on the Schottky barrier formed by the metal/semiconductor contact to control the unidirectional transmission of carriers. To enhance the BV, power SBD structures have incorporated a lightly doped drift layer, as shown in Figure 4. In 2012, Sasaki et al. [36] grew, homogeneously and epitaxially, a 1.4 µm thick epitaxial layer on a β-Ga_2_O_3_ substrate using MBE, fabricating for the first time a Pt/β-Ga_2_O_3_ Schottky barrier diode with a reverse BV exceeding 100 V, a R_on_ of 2 mΩ·cm^2^, and an I_F_ of 200 A/cm^2^ at 1.7 V. In 2015, Higashiwaki et al. [133] first reported a gallium oxide Schottky diode with a 7 µm thick Si-doped n-type drift layer (2.0 × 10^16^ cm^−3^) epitaxially grown using the HVPE technique, featuring a R_on_ of 2.4 mΩ·cm^2^, an ideality factor of 1.02, and a BV approaching 500 V, demonstrating the potential for future applications of β-Ga_2_O_3_-based power devices.

In addition, in high-power circuits there is not only a large voltage but also a large current. Compared with small-area devices, large-area devices are capable of handling higher currents with lower on-state voltage drops. However, the emergence of new materials and new processes inevitably faces the issue of defects; a larger electrode size also means more defects, which significantly impacts the breakdown performance of devices [134,135]. In 2017, Yang et al. [136] fabricated a series of β-Ga_2_O_3_ SBDs with varying electrode sizes. Test results demonstrated that as the electrode size increased, the BV gradually decreased and the R_on_ also reduced. At an electrode diameter of 20 µm, the BV was 1600 V and the R_on_ was 25 mΩ·cm^2^, whereas at 0.53 mm diameter, the BV fell to only 250 V and the R_on_ to 1.6 mΩ·cm^2^.

The crystal structure of β-Ga_2_O_3_ leads to severe anisotropy, which has an important impact on the performance of the device. In 2017, Fu et al. [137] investigated the electrical properties of β-Ga_2_O_3_ SBDs on two distinct crystallographic planes, ($\bar{2}$01) and (010). Findings indicated that the (010) plane had more negative charges and defects on its surface, leading to a more pronounced upward bending of the conduction band. As a result, the R_on_, V_on_, n, and SBH for devices on the (010) plane were higher compared to those on the ($\bar{2}$01) plane, reminding us to consider the anisotropy of the crystal structure in device design.

Furthermore, in 2021, He et al. [138] discovered that β-Ga_2_O_3_ exposed to air over prolonged periods would lead to surface enrichment of donor-like impurities, drastically deteriorating device performance. By removing the unreliable surface layer formed in the air with ICP and preserving the sample in alcohol, minimizing contact with air during the experimental process, they fabricated a device with a R_on_ of 2.25 mΩ·cm^2^ and a BV of 1720 V. The resulting PFOM reached 1.32 GW/cm^2^, making it one of the most outstanding simple-structured β-Ga_2_O_3_ SBD devices to date, fully tapping into the potential applications of gallium oxide in power devices.

#### 4.1.2. Field Plate Structure

**Figure 5:**
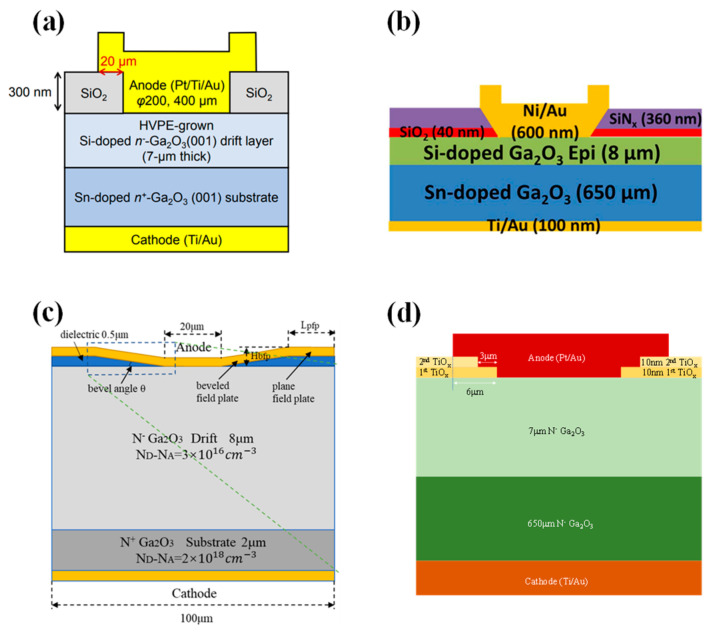
Device schematic of β-Ga_2_O_3_ FP-SBDs with (**a**) SiO_2_ field plate [144]; (**b**) SiO_2_/SiN_x_ bilayer field plate [148]; (**c**) small-angle beveled field plate [149]; (**d**) stair field plate [150].

The field plate structure is to add a layer of dielectric at the edge of the Schottky electrode. By extending part of the electrode, an electric field is formed that acts upon the contact edge to alleviate electric field concentration, thereby effectively increasing the BV of the device. The manufacturing process is simple and controllable, and it is widely applied in SiC and GaN power devices [139,140,141,142]. In 2016, Konishi et al. [143,144] first reported the deposition of SiO_2_ as a field plate structure on β-Ga_2_O_3_ by chemical vapor deposition (CVD), as shown in Figure 5a, where the device’s BV exceeded 1kV. In 2018, Yang et al. [145,146] employed plasma-enhanced chemical vapor deposition (PECVD) to deposit SiN_x_ as a field plate. When the Schottky electrode diameter was 150 µm, the BV reached 2.3 kV and the R_on_ was 0.25 Ω·cm^2^. The reverse recovery time (t_rr_) measured when switching the device from +2 V to −2 V was 22 ns. For devices with Schottky electrode dimensions of 1×1 mm^2^, the I_F_ exceeded 1 A, and the highest BV was 650 V, showcasing the potential application of β-Ga_2_O_3_ SBDs in the high-power domain [147].

Compared to a single-layer field plate, a bilayer field plate can combine the advantages of different dielectrics to further mitigate the electric field crowding effect and improve interface states, thus enhancing device performance. In 2019, Yang et al. employed PECVD to deposit a SiO_2_/SiN_x_ bilayer dielectric as a field plate, as illustrated in Figure 5b, with the SiO_2_ layer capable of absorbing high electric fields and limiting the difference between the conduction and valence bands, effectively improving the breakdown performance of the device. A series of studies was conducted on large-area devices [148,151,152,153]. For devices with a Schottky electrode diameter of 1 mm, the measured BV was 760 V, and the current could reach 1 A at 2.3 V. The t_rr_ from an I_F_ of 1 A to an off-state of −300 V was 64 ns, and it was not affected by a temperature below 150 °C [151]. When the Schottky electrode size was 0.4 × 0.4 mm^2^, the device could also achieve a current of 1 A at 1 V, with a BV of 1900 V and a R_on_ of 0.24 Ω·cm^2^ [152]. The device functionality under a high reverse voltage of −900 V was first demonstrated, with a t_rr_ of 81 ns and a reverse recovery current (I_rr_) of 38 mA [153]. Subsequently, they compared the performance of different bilayer field plate dielectrics (SiO_2_/SiN_x_, Al_2_O_3_/SiN_x_, HfO_2_/SiN_x_) on β-Ga_2_O_3_ FP-SBDs, finding that their forward performance was similar. The Al_2_O_3_/SiNx bilayer field plate exhibited the smallest reverse leakage current, with a BV of 730 V, exceeding that of the SiO_2_/SiN_x_ bilayer field plate at 562 V and the HfO_2_/SiN_x_ bilayer field plate at 401 V [154]. In 2022, Guo et al. [155] fabricated β-Ga_2_O_3_ FP-SBDs with a 1 mm diameter using an Al_2_O_3_/SiN_x_ bilayer field plate, achieving a current of 2 A at 2 V, with a BV of 467 V. The device’s t_rr_ from an I_F_ of 1 A to −100 V was only 8.8 ns, with a reverse recovery charge (C_rr_) of 8.33 nC. With reduced costs and improved material quality, power devices and circuits based on Ga_2_O_3_ are expected to have a broad range of applications in the foreseeable future.

Furthermore, the field plate dielectric significantly impacts device performance. Compared to high-k dielectrics, low-k dielectrics have fewer internal charges and the edge electric field diminishes more rapidly within them, hence exerting a smaller influence on the electric field concentration at the electrode edge. Conversely, in high-k materials, the decrease in electric fields is lower, allowing for more effective propagation of the electric field, which better alleviates the concentration at the electrode edge. In 2021, Roy et al. [156] used a β-Ga_2_O_3_ wafer with an epitaxial layer of only 1.7 µm to deposit an ultra-high-k dielectric BaTiO_3_ (BTO) and BaTiO_3_/SrTiO_3_ (BTO/STO) stack as the field plate structure to fabricate β-Ga_2_O_3_ FP-SBDs. The devices achieved a low R_on_ of 0.32 mΩ·cm^2^, with the BV increasing from 148 V without field plates to 486 V and 687 V, respectively, and a maximum PFOM reaching up to 1.47 GW/cm^2^. Subsequently, BTO was used as the field plate dielectric on a β-Ga_2_O_3_ wafer with an epitaxial layer of 11 µm, resulting in devices with a R_on_ of 6.9 mΩ·cm^2^ and a BV of 2.1 kV for SBDs, which is 2.7 times higher than the structures without field plates, demonstrating the potential of high-k materials as field plate dielectrics [157]. Liu et al. [149], through TCAD simulation studies, found that, under the same reverse bias, the higher the dielectric constant of the field plate dielectrics and the smaller the angle of the beveled field plate, the higher the device BV. This indicates that high-k dielectrics are more effective in mitigating the concentration of the electric field at the electrode edge, and it also points out that the geometry of the field plate has a significant effect on the electric field at the electrode edge. Smaller geometric discontinuities can further alleviate the electric field crowding effect, as in the small-angle beveled field plate illustrated in Figure 5c. Similar to beveled field plates, stair-shaped field plates can also somewhat reduce the geometric abruptness of the field plate. Sun et al. [150] formed a stair-shaped TiO_x_ field plate via the double thermal oxidation of Ti metal, as shown in Figure 5d, increasing the device BV from 460 V to 950 V, while the R_on_ only increased from 2.7 mΩ·cm^2^ to 2.8 mΩ·cm^2^. Kumar et al. [158] fabricated a stair-shaped field plate through PECVD deposition of SiO_2_ and etching processes. Compared to conventional field plate structures, device BVs increased from 980 V to 1530 V. In addition, studies on transistors have shown that the introduction of a field plate structure introduces parasitic capacitance within the device, which results in a decrease in the device cutoff frequency and a deterioration in RF performance, and which is exacerbated by an increase in the dielectric permittivity of the dielectric [159,160,161,162]. However, there are few studies on the effect of introducing the field plate structure on the parasitic capacitance of β-Ga_2_O_3_ diode devices, so it will not be discussed here.

In summary, due to its simple fabrication processes, the field plate structure is widely used in the manufacturing of power devices. By employing composite field plates and high-k dielectrics, and reducing the geometric discontinuities of the device, the concentration at the electrode edges can be further diminished, thus fully capitalizing on the benefits of field plate designs.

#### 4.1.3. Edge Termination Structure

**Figure 6:**
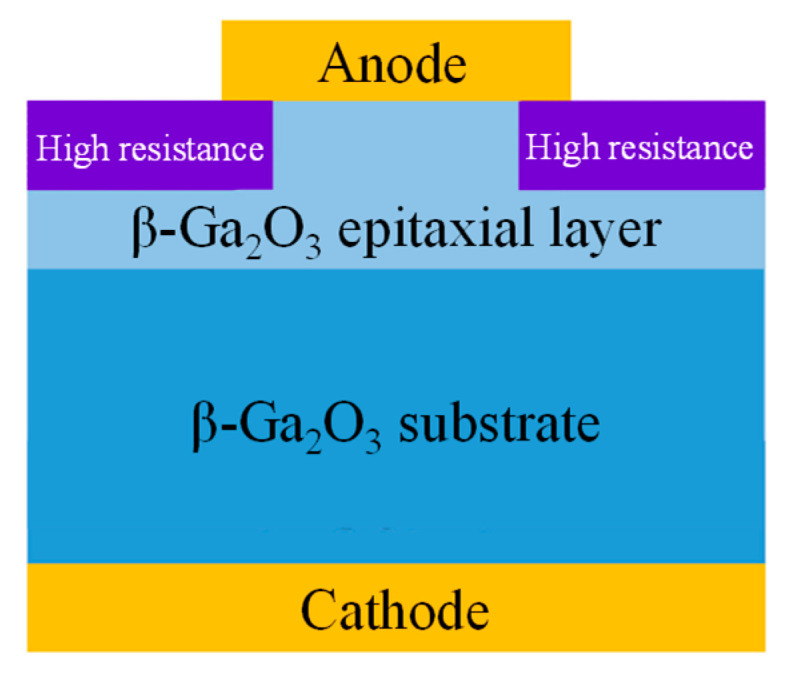
Device schematic of β-Ga_2_O_3_ SBDs with edge termination structure.

Edge termination structure involves the formation of high-resistance regions at the electrode edges through methods such as ion implantation, thermal oxidation, or groove-filling with oxides, resulting in electron isolation, which effectively mitigates electric field crowding at the electrode edges, as shown in Figure 6. Compared to field plate structures, edge terminations reduce geometric discontinuities in devices, but their fabrication processes are more complex. In 2019, Gao et al. [163] reported Ar ion-implanted high-resistance edge-terminated β-Ga_2_O_3_ SBDs; compared to conventional structures, the BV increased from 209 V up to 550 V. In the same year, Lu et al. [164] also reported Ar ion-implanted high-resistance edge terminations. The devices had a R_on_ of 4 mΩ·cm^2^ and an ideality factor of 1.02; compared to traditional structures, the leakage current was reduced by a factor of 10^3^, with an on/off ratio reaching 10^13^, and the BV was enhanced from 257 V to 391 V. Additionally, the devices exhibited excellent dynamic switching performance. Compared to commercial Si fast recovery diodes, the I_rr_ was reduced by a factor of 12 (38 mA), the t_rr_ by 5.5 times (14.1 ns), and the Q_rr_ was only 1.7% (0.34 nC) that of the commercial Si fast recovery diode, fully demonstrating the potential of edge-terminated β-Ga_2_O_3_ SBDs in fast-switching circuits. Zhou et al. [165] achieved β-Ga_2_O_3_ SBD devices with a R_on_ of 5.1 mΩ·cm^2^, an on/off ratio of 10^8^–10^9^, a BV of 1.55 kV, and a PFOM of 0.47 GW/cm^2^ by using Mg ion implantation, thereby demonstrating the advantages of ion-implanted high-resistance edge terminations. However, ion implantation can cause significant damage to the gallium oxide lattice structure, and the heavier the ion mass, the more severe the damage. As a result, a large number of traps and defects are present in the ion-implanted region, leading to device performance degradation under off-state stress [166].

Research indicates that the high-temperature annealing of β-Ga_2_O_3_ in oxygen can facilitate the generation of gallium vacancies, thereby inducing semi-insulating properties [167,168,169,170]. Hence, employing high-temperature oxygen annealing to form high-resistance terminations represents an efficient and low-cost technique. In 2020, Wang et al. [171] utilized SiO_2_ as a barrier layer, forming high-resistance terminations through thermal oxidation at various temperatures, and deposited a SiO_2_ layer atop the high-resistance area as a passivation layer. From C-V measurements, the carrier concentration decreased close to a constant (1 × 10^16^ cm^−3^) after thermal oxidation at higher than 400 °C. Subsequently, the BV increased to a maximum of 940 V, with a R_on_ of 3.0 mΩ·cm^2^ and a PFOM of 295 MW/cm^2^. In 2022, He et al. [172] formed high-resistance terminations using high-temperature thermal oxidation at 1100 °C, employing polycrystalline silicon as the barrier layer. Experiments revealed that the carrier concentration in the polycrystalline silicon area showed no significant depletion (~1.8 × 10^16^ cm^−3^), whereas a pronounced depletion occurred in the exposed region (3.0 × 10^14^ cm^−3^), with a depletion depth of 2.4 µm, indicating the ideal barrier capabilities of polycrystalline silicon against the oxygen-annealing environment. The resulting device exhibited a R_on_ of 4.1 mΩ·cm^2^, a BV of 1800 V, and a PFOM up to 0.78 GW/cm^2^, demonstrating the advantages of thermal oxidation high-resistance termination structures.

Although ion-implanted terminations and thermal oxidation terminations can effectively alleviate electric field crowding, ion-implanted terminations introduce a significant number of traps and defects, and thermal oxidation terminations do not form complete insulating regions, resulting in device BVs that are generally below 2 kV. In 2022, Dong et al. [173] utilized a deep trench filled with a thick SiO_2_ layer to efficiently block current conduction. Since the bandgap of SiO_2_ (8~9 eV) is much larger than that of β-Ga_2_O_3_, it can withstand higher voltages and critical fields, and the relative permittivity of SiO_2_ (~4) is smaller; according to Poisson’s equation, the electric field of the β-Ga_2_O_3_ layer at the interface is less than 1/3 of that of the SiO_2_ layer. Test results showed that the device reached a maximum BV of 6 kV and a minimum R_on_ of 3.4 mΩ·cm^2^, achieving a PFOM of 10.6 GW/cm^2^, surpassing the unipolar limit of SiC and GaN devices and confirming the tremendous potential of edge termination-structured β-Ga_2_O_3_ SBDs as next-generation high-voltage and high-power electronic components.

Moreover, in SiC and GaN devices, another edge termination method is employed, known as the floating metal ring (FMR) structure, which can be fabricated simultaneously with the Schottky contact. This method is simple and achieves effective reverse blocking characteristics [174,175]. Simulation results for β-Ga_2_O_3_ SBD devices also indicate that FMR can effectively increase the device BV, but insufficient experimental verification exists, so it is not discussed in detail here.

To summarize, the edge termination structure is a simple and effective method that can be used to increase the BV by decreasing the electron concentration at the electrode’s edge and reducing the peak value of the electric field. However, the potential of ion implantation termination and thermal oxidation termination structures needs to be explored further through experiments. Additionally, there is a lack of reports on how devices with large-area edge termination structures perform, which needs to be explored in greater detail.

#### 4.1.4. Trench Structure

Although field plate structures and edge termination structures can effectively alleviate electric field concentration at the anode edges and thereby increase the device BV, they rely solely on the Schottky barrier formed by metal–semiconductor contacts to control the reverse blocking of carriers. Under reverse bias, a high reverse electric field exists near the Schottky contact interface, leading to a large reverse leakage current [176]. In an effort to diminish the leakage current and regulate the field strength distribution from the Schottky contact interface to the interior of the device, the trench structure for SBDs can be a promising choice; under reverse bias, the trench’s metal oxide semiconductor (MOS) structure depletes surface charges and reduces the surface electric field (RESURF), reducing the leakage current path. At high reverse biases, it can even pinch off the trench channel, effectively decreasing the leakage current and enhancing the device BV [177,178,179].

**Figure 7:**
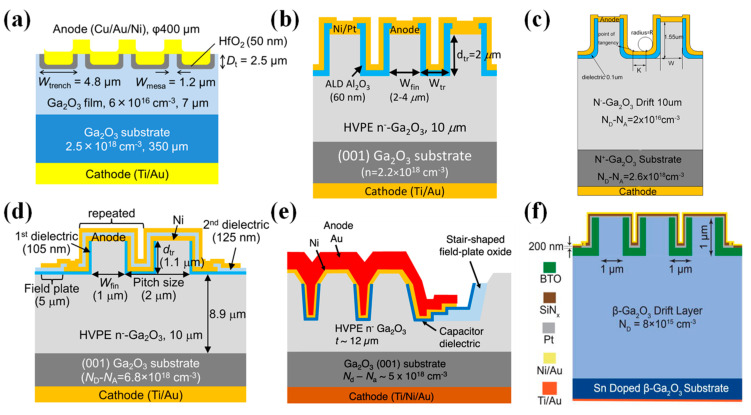
Device schematic of β-Ga_2_O_3_ trench MOS SBDs with (**a**) 7 µm drift layer [181]; (**b**) 10 µm drift layer [183]; (**c**) trench bottom corner [187]; (**d**) edge field-plated structure [188]; (**e**) edge stair-shaped field-plated structure [189]; (**f**) BaTiO_3_ dielectric [190].

In 2017, Sasaki et al. [180,181] prepared β-Ga_2_O_3_ trench MOS SBDs for the first time on β-Ga_2_O_3_ substrates with a 7 µm drift layer (6 × 10^16^ cm^−3^), as illustrated in Figure 7a. The experiments demonstrated that the trench MOS SBDs are effective in reducing the reverse leakage current, increasing the BV from 70 V to 240 V. Due to the reduced current channel, the R_on_ increased from 2.3 mΩ·cm^2^ to 2.9 mΩ·cm^2^. Subsequently, the reverse recovery characteristics of the devices were examined through a double-pulse test circuit, going from an I_F_ of 1 A to a reverse bias of 100 V. The I_rr_ was measured at 0.42 A, with a t_rr_ of 7.6 ns, and a recovery loss of 0.12 µJ. These performances are nearly on par with commercial SiC SBDs [182]. 

The device structure and preparation process substantially affect performance, including the epitaxial layer thickness, doping concentration, fin width, fin orientation, and etching processes. In 2018, Li et al. [183] developed β-Ga_2_O_3_ trench MOS SBDs on a β-Ga_2_O_3_ substrate with a 10 µm drift layer (1–2 × 10^15^ cm^−3^), depicted in Figure 7b, achieving a BV of 1.5 kV, and a four-magnitude decrease in the reverse leakage current relative to conventional SBDs. Further experimentation on substrates with drift layer concentrations of 2 × 10^16^ cm^−3^ resulted in similar devices with a BV of 1232 V, indicating that an increased epitaxial layer doping concentration can lead to a reduction in BV. Furthermore, the results indicated that a reduced fin width enhanced the RESURF effect, leading to a lower leakage current and higher BV [184]. In addition, they discovered that devices aligned along the (001) direction exhibited the highest forward current, while other orientations, due to interface negative charges, experienced severe sidewall conduction losses, causing shallow turn-on behavior and a substantial reduction in forward current [185]. In the same year, during the IEDM conference, they reported β-Ga_2_O_3_ vertical trench SBDs with a BV of 2.44 kV by introducing wet-etching after dry-etching to minimize the etching damage; for devices with fin widths of 1–2 µm, the reverse leakage current density remained below 1 µA/cm^2^ up to the BV, which consistently occurred at the trench corners, where electric field crowding is prone to happen [186].

To mitigate the electric field crowding at the trench bottom corners, Huang et al. [187] introduced a bottom corner structure, as shown in Figure 7c, and optimized its structural parameters through TCAD simulations. Simulation results indicated that the maximum BV could reach 3.4 kV, with a PFOM as high as 2 GW/cm^2^. Besides the trench bottom corners, electric field crowding also occurs at the edges of the trench region. Li et al. [188] introduced a field-plated structure at the trench region edges by twice depositing Al_2_O_3_ dielectric, as illustrated in Figure 7d. Compared to trench MOS devices without a field plate structure, the BV increased from 2.4 kV to 2.9 kV, and the PFOM reached 0.95 GW/cm^2^, demonstrating the efficacy of the field plate structure in alleviating electric field crowding at the trench region edges. Otsuka et al. [189] fabricated large-area (1.7 × 1.7 mm^2^) β-Ga_2_O_3_ trench MOS SBDs by introducing a stair-shaped field plate structure via SiO_2_ deposition and double wet-etching at the edges of the trench region, as depicted in Figure 7e. The devices displayed an I_F_ of 2 A at 2 V, and the reverse leakage current was only 5.7 × 10^−10^ A at a reverse bias of −1.2 kV, achieving an on/off ratio surpassing 10^9^. Additionally, Roy et al. [190] used the ultra-high-k dielectric material BaTiO_3_ as the dielectric and introduced a SiN_x_-passivated dielectric layer to fabricate trench MOS SBDs of varying areas, as shown in Figure 7f. Small-area devices (200 × 200 µm^2^) exhibited a BV exceeding 3 kV, and the reverse leakage current remained below 1 µA/cm^2^ at 3 kV. For large-area devices, devices measuring 1 × 1 mm^2^ and 2 × 2 mm^2^ had BVs of 1.8 kV and 1.4 kV, respectively, and could sustain a forward current of 3.7 A and 15 A under a 10% duty cycle pulse test. Moreover, the device characteristics, such as the temperature coefficient of resistance, capacitance, stored charge, and switching energy ratio, were smaller compared to commercially available SiC SBDs of equivalent ratings, showcasing the advantages of β-Ga_2_O_3_ trench MOS SBDs for high-power applications.

Dry-etching has been found to introduce a significant number of defects at the trench sidewall interfaces, substantially impacting device performance. Li et al. [191] observed that the density of negatively charged traps at the sidewall interface increases with a rising forward voltage, and the release of captured charges is very slow at room temperature, resulting in sidewall depletion, which leads to current collapse and delayed turn-on behavior. To mitigate the defects caused by dry-etching, Tang et al. [192] treated devices post-dry-etching with self-reactive etching (SRE) in an MBE, where metal Ga reacts with Ga_2_O_3_ to form Ga_2_O, and the suboxide Ga_2_O is gas, which can be discharged with the exhaust gas to achieve etching purposes. After SRE treatment, a smooth surface with the same morphology as the original substrate can be obtained, and the extremely low interfacial density of states of the device (2.9 × 10^11^ cm^−2^·eV^−1^) confirms the repairing effect of the SRE process on the etched surface. Moreover, the device boasted excellent thermal stability, with a forward current density of 1228 A/cm^2^ at 3 V and an on/off ratio surpassing 10^10^ [193]. Following this, Dhara et al. [194] employed a similar approach to fabricate β-Ga_2_O_3_ trench MOS SBDs, achieving a BV of 1.45 kV, a R_on_ of 1.20 mΩ·cm^2^, and a PFOM exceeding 2 GW/cm^2^, attesting to the effectiveness of the SRE process.

Overall, although the presence of the trench reduces the device’s effectiveness, the conduction area is reduced, resulting in a lower I_F_, and a larger V_on_ due to the depletion of the negative charge at the trench sidewall interface. However, among the various structures of β-Ga_2_O_3_ SBDs, the trench structure has the lowest reverse leakage current, which results in a very low off-state loss and high breakdown voltage, and thus β-Ga_2_O_3_ trench MOS SBDs also have a large potential for application in high-power devices.

#### 4.1.5. Mesa Termination Structure

**Figure 8:**
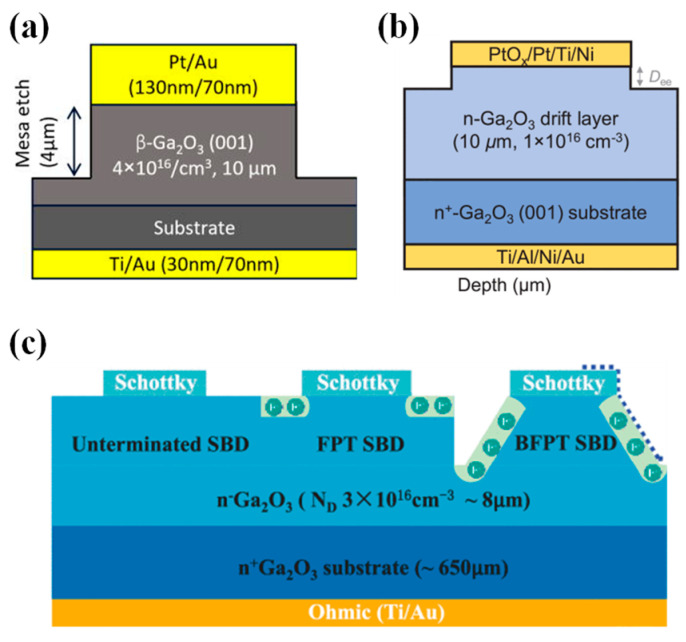
Device schematic of mesa termination structure β-Ga_2_O_3_ SBDs with (**a**) 4 µm deep mesa [195]; (**b**) PtO_x_ Schottky electrode [196]; (**c**) beveled F plasma-treated (BFPT) structure (right) [197].

The mesa termination structure, achieved through an etching process that raises the Schottky contact area, reduces the geometrical abruptness of the Schottky contact region, which is transferred to the inside of the β-Ga_2_O_3_ material to alleviate the electric field concentration at the electrode edges and improve device performance. The fabrication process of the basic mesa structure is very simple, with only one additional step of the self-aligned etching process compared to simple β-Ga_2_O_3_ SBDs. As depicted in Figure 8a, Dhara et al. [195] employed a Pt metal mask to create a 4 µm deep mesa structure using inductively coupled plasma (ICP)-etching, which increased the BV of the device from 350 V to 1150 V when compared to a simple structure. Han et al. [196], utilizing a PtO_x_ Schottky electrode as shown in Figure 8b, demonstrated that, when the mesa etching depth was set at 1.2 µm, the device achieved a BV of 2738 V and a PFOM of 1.02 GW/cm^2^. Moreover, this device maintained a leakage current density of less than 10 µA/cm^2^ up to −2000 V, thereby manifestly showcasing the application potential of PtO_x_ Schottky electrodes and mesa structure for β-Ga_2_O_3_ power devices.

Moreover, Hu et al. [197] introduced F ions on the surface through plasma treatment, to alleviate the electric field concentration by utilizing the strong electronegativity of F ions to attract negative charges to gather near the surface, as shown in Figure 8c. Compared to a device without any terminal structures, the F plasma-treated (FPT) device exhibited an increase in BV from 250 V to 520 V. The incorporation of a beveled F plasma-treated (BFPT) structure further enhanced the BV up to 1050 V, indicating that a mesa structure can effectively alleviate electric field concentration effects. Wei et al. [198] introduced a thermal oxidation treatment (TOT) process within the mesa structure to reduce the surface electron concentration, passivate the oxygen vacancy-type interface states to improve device reliability, and deposit a SiO_2_ passivation layer to prepare β-Ga_2_O_3_ SBDs with a Schottky contact size of 2 × 2 mm^2^. The devices achieved a BV of 600 V, with reverse leakage currents maintained below 10 µA and a forward current capability of up to 7 A. Exhibiting commendable thermal stability during a high-temperature storage (HTS) test at 450 K, these findings underscore a formidable potential for high-temperature, high-power applications.

**Figure 9:**
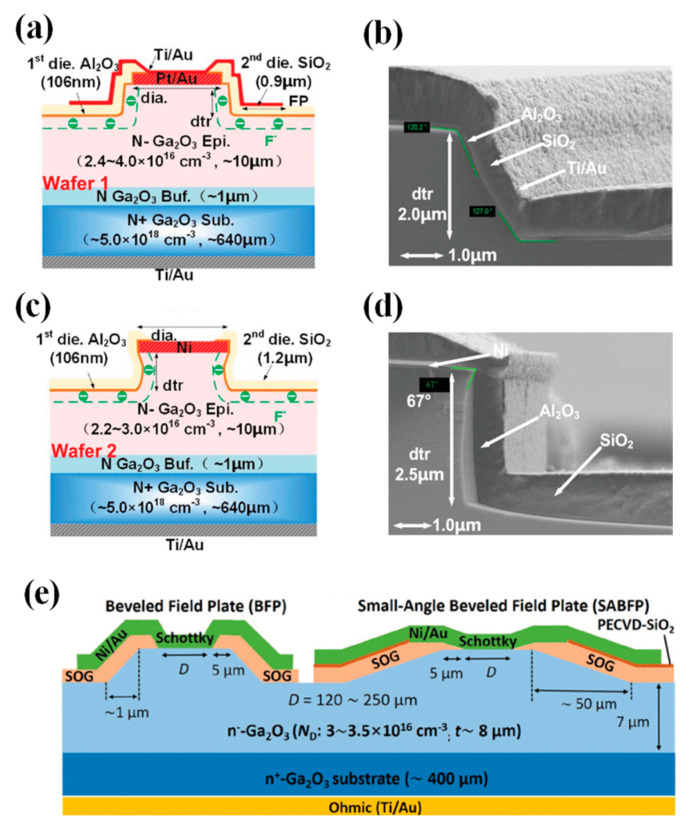
Device schematic of (**a**) FP-NB SBD and (**b**) SEM cross-section image; (**c**) PB SBD and (**d**) SEM cross-section image [199]; (**e**) β-Ga_2_O_3_ BFP and SABFP SBDs [200].

Similar to the trench structure, the mesa structure can also improve the surface electric field by introducing a side MOS structure. The inclination angle of the mesa has an important effect on the performance of the device. Chen et al. [199] used SiO_2_ and Ni as etching masks to fabricate negative- and positive-beveled mesa structures, respectively. After F plasma treatment and the deposition of bilayer field plate dielectrics, they constructed SBDs as shown in Figure 9a–d. Compared to planar SBDs, the negative-beveled (NB) mesa structure increased the BV from 400 V to 1100 V; the positive-beveled (PB) mesa structure proved even more effective in mitigating the electric field concentration, achieving a BV of 1710 V. However, due to the reduced conduction area, the R_on_ of the PB device increased to 3.6 mΩ·cm^2^, with a PFOM of 0.8 GW/cm^2^. Allen et al. [200] successfully fabricated a small-angle beveled mesa structure with a 1° tilt, using a dual-mask wet-etching technique. They created β-Ga_2_O_3_ SBDs incorporating a bilayer field plate using PECVD-SiO_2_/spin-on glass (SOG), as illustrated in Figure 9e, and compared the BV across devices with simple structures, ordinary field plate structures, 45° beveled field plate (BFP) structures, and 1° small-angle beveled field plate (SABFP) structures, which were approximately ~200 V, ~400 V, ~650 V, and ~1100 V respectively. This indicates that SABFP can more effectively alleviate the concentration of electric fields.

In summary, the simple mesa structure is an effective and straightforward approach for mitigating electric field concentration at the electrode edges, which significantly enhances device performance. Mesa structures equipped with field plates are similar to trench structures and offer a larger conduction area, hence providing superior forward performance. Consequently, they hold considerable potential for application in β-Ga_2_O_3_ power devices.

### 4.2. Vertical Heterojunction-Structure Diodes

Although SBDs exhibit a lower V_on_ and faster recovery times compared to p-n junction diodes, and the BV of SBDs is greatly improved due to the wide bandgap of β-Ga_2_O_3_ and the introduction of various termination structures, it is still challenging to see their BV exceed 3 kV, and the fabrication processes involved are more complex. P-n junction diodes can not only greatly improve the BV of the device and exhibit lower leakage currents, but they can also effectively reduce the R_on_ through the modulation of electrical conductivity [201]. However, the absence of an effective p-type Ga_2_O_3_ has hindered the development of β-Ga_2_O_3_ p-n homojunction diodes. Researchers have thus turned their attention to constructing β-Ga_2_O_3_ p-n heterojunction diodes (HJDs), using other p-type materials to achieve Ga_2_O_3_ bipolar power devices. These include Cu_2_O [202], NiO [203,204], GaN [205,206], SnO [207], CuAlO_2_ [208], and others. Notably, NiO has a bandgap of 3.8–4 eV, controllable doping characteristics, and a hole mobility of 0.5–5 cm^2^/V·s, which can form a type II band alignment with β-Ga_2_O_3_ [209,210]. Currently, the majority of β-Ga_2_O_3_ p-n heterojunction devices utilize NiO and have achieved significant breakthroughs in performance. This section primarily summarizes the NiO/β-Ga_2_O_3_ p-n HJDs, heterojunction barrier Schottky (HJBS) diodes, and junction termination extension (JTE) structures.

#### 4.2.1. Heterojunction Diodes

**Figure 10:**
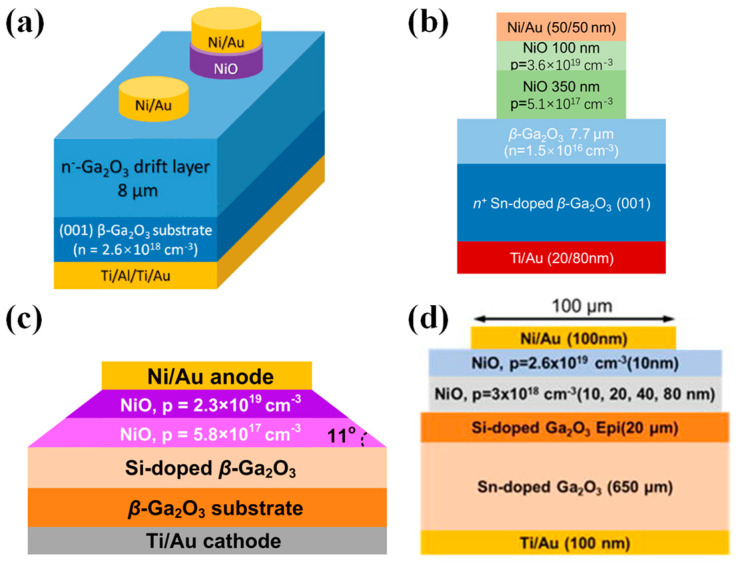
Device schematic of (**a**) NiO/β-Ga_2_O_3_ HJDs and Ni/β-Ga_2_O_3_ SBDs [201]; (**b**) NiO/β-Ga_2_O_3_ HJDs with bilayer p-NiO [215]; (**c**) beveled mesa NiO/β-Ga_2_O_3_ HJDs [219]; (**d**) NiO/β-Ga_2_O_3_ HJDs with JTE [221].

P-type NiO thin films can be fabricated by several methods, including the sol–gel process [211], electron beam evaporation (EBE) [212], sputtering [213], and thermal oxidation [214]. Among these, sputtering is widely used due to its rapid growth rate and ability to control the doping concentration of NiO films by adjusting the inflow ratio of oxygen. In 2020, Lu et al. [201] reported a practical NiO/β-Ga_2_O_3_ HJDs by sputtering a 200 nm NiO layer with a net doping concentration of 4 × 10^16^ cm^−3^ onto β-Ga_2_O_3_, as shown in Figure 10a, exhibiting a R_on_ of 3.5 mΩ·cm^2^, lower than that of the Ni/β-Ga_2_O_3_ SBDs (4.2 mΩ·cm^2^). The BV was improved from 500 V to 1059 V, with a leakage current below 1 µA/cm^2^ before breakdown. Subsequently, Gong et al. [215] fabricated HJDs with a bilayer NiO structure, as demonstrated in Figure 10b, where the high-doped layer provides a high hole concentration, and the low-doped layer effectively suppresses electric field crowding. Compared to the single-layer device with a BV of 0.94 kV, the double-layer device achieved a BV of 1.86 kV. Moreover, experiments have shown that post-annealing treatment can significantly reduce the trap density at the NiO/β-Ga_2_O_3_ interface and improve device performance [216,217]. Hao et al. obtained a device with a BV of 2.66 kV and a PFOM of 2.83 GW/cm^2^ through annealing, which also exhibited excellent thermal stability, maintaining a BV of 1.77 kV at 250 °C [218].

Similar to mesa structures, HJDs can also reduce the geometrical abruptness between p-n junctions through a small-angle structure to alleviate the electric field concentration and improve the device’s performance. Zhou et al. [219] fabricated a large-area (1 × 1 mm^2^) small-angle (~11°) beveled mesa NiO/β-Ga_2_O_3_ HJD, as illustrated in Figure 10c. The device exhibited a static BV of 1.95 kV and a dynamic BV of 2.23 kV, with an I_F_ reaching 20 A and a R_on_ of 1.9 mΩ·cm^2^. The subsequent fabrication of HJDs with an inclined angle of 6° resulted in a BV of up to 2.04 kV. Under DC (pulsed) conditions, the device’s R_on_ was measured at 2.26 (1.45) mΩ·cm^2^, with a PFOM of 1.84 (2.87) GW/cm^2^. The device demonstrated a t_rr_ of 16.4 ns under switching conditions of 800 V/10 A and exhibited high thermal stability at 200 °C, due to thermally enhanced conductance modulation [220].

Experimental and simulation studies indicate that the extension of the NiO layer beyond the edge of the metal contacts, as depicted in Figure 10d, produces a protective ring effect that can disperse the electric field crowding at the edges of the diode [221,222,223]. Li et al. [221] fabricated a 100 µm diameter NiO/β-Ga_2_O_3_ HJD on a wafer with a 20 µm epitaxial layer (2 × 10^16^ cm^−3^). The device achieved a BV of 4.7 kV and a R_on_ of 11.3 mΩ·cm^2^, with a PFOM reaching 2 GW/cm^2^. Subsequently a large-area HJD, with a 1 mm diameter, demonstrated a R_on_ of 11.86 mΩ·cm^2^ and a BV to 1.76 kV. The device featured a t_rr_ of 101 ns when switched from an I_F_ of 1 A to a reverse bias of −550 V [224].

Further, the low-power sputtering of NiO was used to reduce the interfacial damage at the heterointerface on β-Ga_2_O_3_ with a doping concentration of the drift layer less than 10^16^ cm^−3^, while the diameter of the NiO layer was larger than the Schottky electrodes to form a protective ring. With a Schottky electrode diameter of 100 µm, p-NiO/β-Ga_2_O_3_ HJDs with a maximum BV of 8.9 kV were achieved, exhibiting a R_on_ of 7.9 mΩ·cm^2^ and a PFOM surpassing 10 GW/cm^2^. Devices with an area of 1 mm^2^ also reached a BV of 4.7 kV and an I_F_ of 4.1 A at 10 V, with a PFOM of 9 GW/cm^2^ [225,226,227]. The performance of both small-area and large-area devices exceeded the limit of unipolar power devices based on SiC and GaN, fully demonstrating the potential of β-Ga_2_O_3_ in future high-power applications.

**Figure 11:**
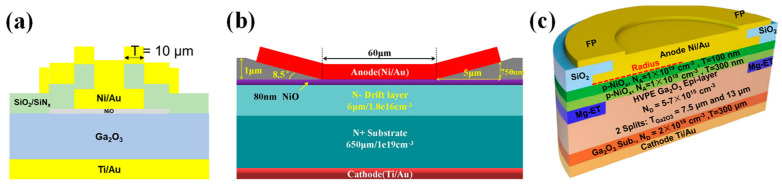
Device schematic of NiO/β-Ga_2_O_3_ HJDs with (**a**) SiN_x_/SiO_2_ bilayer field plate structure [228]; (**b**) small-angle BFP [229]; (**c**) Mg ion implantation terminals and SiO_2_ field plate structure [39].

The performance of HJDs can be further enhanced through the introduction of additional terminal structures. Li et al. [228] prepared a large-area (1 × 1 mm^2^) NiO/β-Ga_2_O_3_ HJD on β-Ga_2_O_3_ with a 15 µm drift layer, simultaneously introducing a SiO_2_/SiN_x_ bilayer field plate structure, as portrayed in Figure 11a. Compared to the device without the field plate structure, the BV increased from 5 kV to 7 kV, and the PFOM improved from 5.7 to 9.2 GW·cm^−2^. Wang et al. [229] applied a photoresist reflow technique to introduce a field plate structure with a small angle (~8.5°), as shown in Figure 11b. Compared to the vertical field plate structure, the BV of the device increased from 1945 V to 2410 V, and the device exhibited a R_on_ of just 1.12 mΩ·cm^2^, enabling a PFOM up to 5.18 GW/cm^2^. Zhang et al. [39] simultaneously used Mg ion implantation terminals and a SiO_2_ field plate structure to mitigate the effect of the electric field concentration of the NiO/β-Ga_2_O_3_ heterojunction, as displayed in Figure 11c. The device achieved a BV of up to 8.32 kV, a R_on_ of 5.24 mΩ·cm^2^, and a PFOM as high as 13.2 GW/cm^2^. This surpasses the unipolar limit of gallium nitride and silicon carbide, demonstrating its tremendous potential in next-generation power electronics applications.

Metal–dielectric–semiconductor (MDS) HJDs were fabricated by inserting an insulating dielectric layer between the metal and β-Ga_2_O_3_, effectively enhancing the device’s reverse characteristics while generally observing a decrease in forward performance, and they will not be discussed in detail here [230,231,232,233,234].

#### 4.2.2. Heterojunction Barrier Schottky Diodes

**Figure 12:**
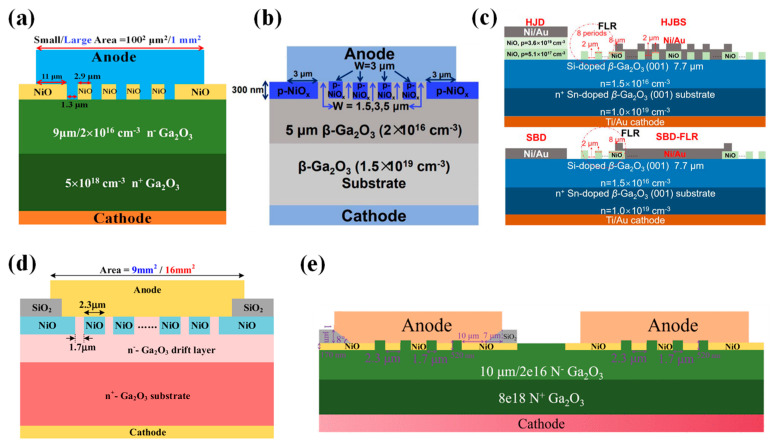
Device schematic of (**a**) β-Ga_2_O_3_ HJBS diodes with thermally oxidized p-NiO [235]; (**b**) β-Ga_2_O_3_ HJBS diodes with sputter-deposited p-NiO [236]; (**c**) NiO/β-Ga_2_O_3_ HJDs, HJBS-2 µm diodes terminated with p-NiO FLRs, Ni/β-Ga_2_O_3_ SBDs, and Ni/β-Ga_2_O_3_ SBDs terminated with p-NiO FLRs [237]; (**d**) β-Ga_2_O_3_ HJBS diodes with SiO_2_ field plate [238]; (**e**) β-Ga_2_O_3_ HJBS diodes with beveled field plate structure (**left**) and without beveled field plate (**right**) [239].

Although HJDs exhibit a higher BV, their junction capacitance and reverse recovery time are increased due to carrier recombination. To combine the advantages of p-n junctions and SBDs, researchers have proposed heterojunction barrier Schottky (HJBS) diodes. In 2020, Lv et al. [235] reported the first realization of β-Ga_2_O_3_ HJBS diodes through the thermal oxidation of p-NiO, as shown in Figure 12a. The diodes with an area of 100 × 100 μm^2^ achieved a BV of 1715 V and a Ron of 3.45 mΩ∙cm^2^, yielding a PFOM of up to 0.85 GW/cm^2^. Additionally, large-scale HJBS diodes with an area of 1 × 1 mm^2^ reached an I_F_ and BV of 5 A/700 V. Yan et al. [236] fabricated HJBS diodes by sputter-depositing p-NiO in a β-Ga_2_O_3_ groove, as illustrated in Figure 12b. With a fin width of 3 µm, the device exhibited a R_on_ of only 1.94 mΩ∙cm^2^ and a BV of 1.34 kV, leading to a PFOM of 0.93 GW/cm^2^. Furthermore, the sidewall depletion effect of the p-NiO was enhanced with a reduction in fin width, resulting in a decreased reverse leakage current. 

Gong et al. [237] compared five different device structures, including HJBS diodes with field limiting rings (FLRs) of 2 µm or 3 µm, a 2 µm FLR Ni/β-Ga_2_O_3_ SBD, a Ni/β-Ga_2_O_3_ SBD without edge termination, and a NiO/β-Ga_2_O_3_ HJD, as depicted in Figure 12c. The HJBS-2 µm device demonstrated the highest BV of 1.89 kV, a R_on_ of 7.7 mΩ∙cm^2^, and a PFOM of 0.46 GW/cm^2^, with the reverse leakage mechanism identified as Poole–Frenkel emission according to J-V-T measurements.

The integration of field plate structures can further enhance the performance of HJBS diodes. Wei et al. [238] fabricated β-Ga_2_O_3_ HJBS diodes with dimensions of 3 × 3 mm^2^ and 4 × 4 mm^2^ through thermal oxidation of p-NiO, incorporating SiO_2_ field plates, as shown in Figure 12d. The BV and specific R_on_ were measured at 550 V/500 V and 11 mΩ·cm^2^/15 mΩ·cm^2^, respectively, with the current exceeding 50 A under a forward bias of 6 V. Long-term high-temperature stress tests showed that the devices had good electrical and thermal reliability. Wu et al. [239] introduced a small-angle (~8°) SiO_2_ field plate structure on the edge of the HJBS diodes, as depicted in Figure 12e, which increased the BV of the small-scale (0.1 × 0.1 mm^2^) diode from 1895 V to 2395 V, achieving a PFOM of 0.72 GW/cm^2^. For the large-scale (3 × 3 mm^2^) diode, the BV rose from 685 V to 1060 V, and after packaging, the device exhibited a t_rr_ of 26.8 ns under switching conditions, with a di/dt of 400 A/µs. These findings demonstrate the significant potential for β-Ga_2_O_3_ HJBS diodes in various applications.

**Figure 13:**
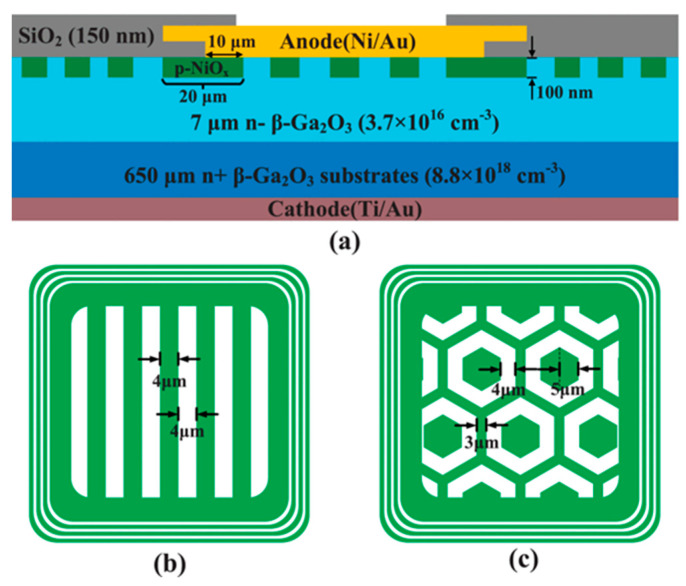
(**a**) Schematic cross-sectional diagram of NiO_x_/β-Ga_2_O_3_ HJBS diodes; (**b**) schematic (top views) of anode layout of stripe HJBS diodes and (**c**) honeycomb HJBS diodes. (The green regions are p-NiO_x_ and the white regions are Ga_2_O_3_.) [240].

The distribution of p-NiO also has a significant impact on the performance of HJBS diodes. Zhang et al. [240] compared the performance of 1 mm^2^ NiO_x_/β-Ga_2_O_3_ HJBS diodes with stripe and honeycomb anode island layouts, as shown in Figure 13. In comparison to the stripe HJBS diodes, the honeycomb HJBS diodes had a slightly higher V_on_ and R_on_ but a BV that increased from 412 V to 567 V, resulting in a 59% increase in BFOM. Furthermore, the honeycomb HJBS diodes exhibited superior surge current stability when compared to the stripe HJBS diodes, attributed to the superior heat dissipation capability of the honeycomb layout. Simulated results suggested that reducing the size of the honeycomb could further enhance its forward conduction capability.

#### 4.2.3. Junction Termination Extension Structures and Super Junction SBDs

**Figure 14:**
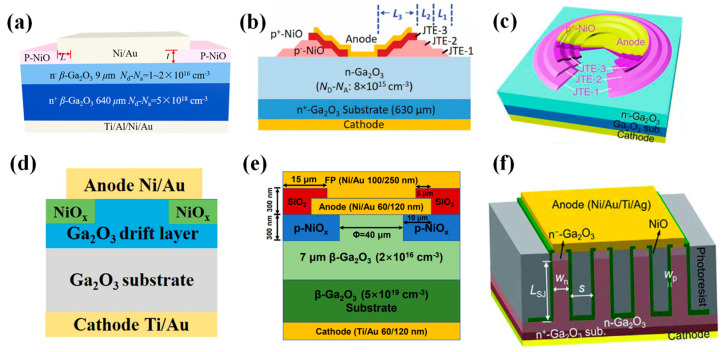
Device schematic of (**a**) β-Ga_2_O_3_ SBDs with p-NiO JTE [242]; (**b**) β-Ga_2_O_3_ SBDs with NiO stair-shaped JTE [243]; (**c**) NiO/β-Ga_2_O_3_ HJDs with NiO stair-shaped JTE [244]; (**d**) β-Ga_2_O_3_ SBDs with p-NiO_x_ edge termination extension structure [245]; (**e**) β-Ga_2_O_3_ SBD with edge termination extension and field plate structure [246]; (**f**) β-Ga_2_O_3_ SJ-SBDs [247].

By introducing p-NiO into various termination structures to replace conventional field plate dielectrics, devices can benefit from the conductance modulation effect to effectively reduce the R_on_, while lateral expansion of the junction termination can alleviate electric field crowding and enhance device performance. In 2022, Hao et al. [241,242] utilized p-NiO to form junction termination extension (JTE) structures, as shown in Figure 14a. The resulting devices exhibited a R_on_ of 2.9 mΩ∙cm^2^, a BV of 2.11 kV, and a PFOM reaching 1.54 GW/cm^2^. Additionally, they fabricated large-area SBDs with an area of 0.78 mm^2^, achieving a forward current density of 180 A/cm^2^ at 2 V, a BV of up to 1.3 kV, and measuring a t_rr_ of 15.6 ns and C_rr_ of 15.3 nC, on par with commercial SiC SBDs. Wang et al. [243] constructed stair-shaped JTE structures comprising multiple layers of p-NiO, as depicted in Figure 14b; the device showed a R_on_ of 5.9 mΩ∙cm^2^, a BV of greater than 2.5 kV, and a PFOM surpassing 1 GW/cm^2^. Xiao et al. [244] introduced a stair-shaped JTE structure on the edge of a NiO/β-Ga_2_O_3_ HJD, illustrated in Figure 14c, improving the device’s BV from 1770 V to 3590 V, with a PFOM reaching 2.7 GW/cm^2^. These developments convincingly demonstrate the potential for p-NiO termination extension structures in β-Ga_2_O_3_ power devices.

Integrating p-NiO within the edge termination structure also enables the formation of an edge termination extension structure, as depicted in Figure 14d [245]. Compared to a simple structure, the BV of the device was increased from 356 V to 1539 V, and p-NiO_x_ effectively passivated the damage caused by dry-etching, yielding ideality factors close to 1 across varying temperatures, albeit with a notable reduction in forward current. As the temperature increases, the hole concentration increases, resulting in a lower leakage current of the SBDs compared to conventional SBDs at high temperatures, and the small polarization transport model in NiO_x_ is used to explain this phenomenon. Yan et al. [246] fabricated p-NiO_x_ edge termination structures with integrated SiO_2_ field plates, as shown in Figure 14e. For Schottky electrodes with a diameter of 60 µm, the device achieved a maximum BV of 2000 V, a R_on_ of 3.12 mΩ∙cm^2^, and a PFOM of 1.11 GW/cm^2^. The same process was utilized to create large-area SBDs with a diameter of 1240 µm, which exhibited a high I_F_ of 7.13 A at 4.9 V, a BV of 1260 V, and a PFOM of 235 MW/cm^2^. 

Qin et al. [247] successfully fabricated vertical super junction Schottky barrier diodes (SJ-SBDs) by integrating p-NiO within the trenches of β-Ga_2_O_3_, as illustrated in Figure 14f. They employed bilayer β-Ga_2_O_3_ epitaxial growth to realize a low R_on_, used a SiO_2_ sacrificial layer to prevent etching damage to the NiO, and controlled the parameters of the NiO layer for charge balance. The resulting devices exhibited a R_on_ of only 0.7 mΩ·cm^2^, a BV reaching 2000 V, and maintained a BV in excess of 1.8 kV at 175 °C. Device robustness was confirmed under dynamic voltage conditions, withstanding breakdown at 2.2 kV in unclamped inductive switching (UIS) tests. Furthermore, the SJ-SBDs demonstrated no parameter changes after hundreds of cycles at a peak voltage of 1.7 kV during repetitive UIS tests, validating the device’s robust functionality under continuous switching.

In summary, β-Ga_2_O_3_ p-n heterojunction devices are the most investigated to date and exhibit superior overall performance, making them highly promising for power circuit applications. In the future, a further advancement in p-n junction devices is anticipated with the effective doping of p-type β-Ga_2_O_3_.

### 4.3. Lateral-Structure Diodes

With the swift advancement of vertical β-Ga_2_O_3_ SBDs, lateral β-Ga_2_O_3_ SBDs have also seen considerable development. Although lateral structures require a larger area compared to their vertical counterparts, they can be integrated with heterogeneous substrates to significantly reduce costs and improve thermal dissipation, mitigating the self-heating effects due to the low thermal conductivity of β-Ga_2_O_3_ materials. This section predominantly summarizes lateral β-Ga_2_O_3_ SBDs with a BV exceeding 1 kV.

**Figure 15:**
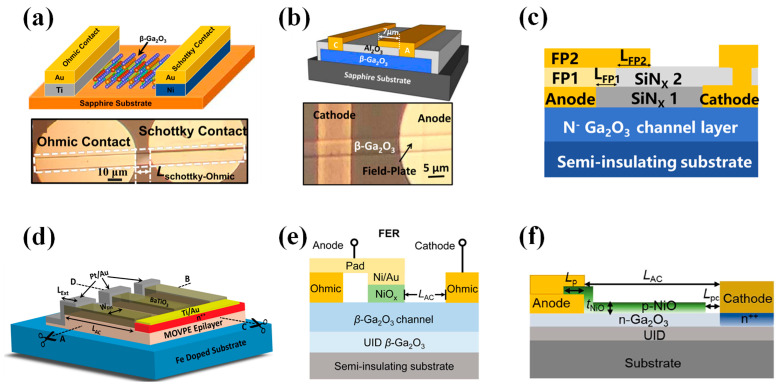
Device schematic of (**a**) lateral β-Ga_2_O_3_ SBDs on sapphire substrate and top-view microscopy image [248]; (**b**) lateral β-Ga_2_O_3_ SBDs with field plate structure and top-view microscopy image [249]; (**c**) lateral β-Ga_2_O_3_ SBDs with bilayer field plate [38]; (**d**) β-Ga_2_O_3_ lateral super junction SBDs with BaTiO_3_ dielectric [250]; (**e**) β-Ga_2_O_3_ field-effect rectifier with a p-NiO_x_ gate [251]; (**f**) lateral β-Ga_2_O_3_ RESURF SBDs [37].

In 2018, Hu et al. [248] transferred a β-Ga_2_O_3_ nano-membrane onto a sapphire substrate via mechanical exfoliation, producing simple-structured lateral SBDs as depicted in Figure 15a. With an anode–cathode spacing (L_AC_) of 15 µm, these devices achieved a BV of 1.7 kV, although the breakdown field was only 1.13 MV/cm and the R_on_ was relatively high at 190 mΩ·cm^2^. Even at 150 °C, the on/off ratio remained over 10^7^, demonstrating the cooling advantage of the sapphire substrate. In the same year, by integrating a field plate structure as shown in Figure 15b, they developed lateral β-Ga_2_O_3_ SBDs with a reduced R_on_ of 10.2 mΩ·cm^2^, an enhanced BV of 2.25 kV, and a PFOM of 500 MW/cm^2^ when the L_AC_ = 16 µm [249]. Wang et al. [38] employed a bilayer field plate structure and introduced a post-anode annealing (PAA) technique to refine the metal/β-Ga_2_O_3_ interface, as shown in Figure 15c. With a L_AC_ = 90 μm, they achieved lateral β-Ga_2_O_3_ SBDs with a BV surpassing 10 kV and a R_on_ of 485 mΩ·cm^2^.

Roy et al. [250] epitaxially grew a Si-doped β-Ga_2_O_3_ thin film on Fe-doped β-Ga_2_O_3_ insulating substrates by MOCVD. They etched the film into rectangular trenches using RIE and utilized the ultra-high-k material BaTiO_3_ as the dielectric to construct lateral super junction structures, as depicted in Figure 15d. The devices achieved a maximum BV of 2359 V, with a L_AC_ = 5 μm and fin width of 2 μm resulting in a BV of 1487 V and a R_on_ of only 1.65 mΩ·cm^2^, demonstrating a PFOM reaching 2.7 GW/cm^2^ and effectively showcasing the superiority of the lateral super junction structure.

The introduction of p-NiO also significantly enhanced the performance of lateral structure devices. Liu et al. [251] proposed a β-Ga_2_O_3_ field-effect rectifier (FER) featuring a p-NiO_x_ gate, as illustrated in Figure 15e. Compared to the lateral HJDs and SBDs on the same substrate, the p-NiO_x_ provided an additional conductive path under high forward bias, resulting in the highest I_F_ and the lowest R_on_, with the V_on_ being only 41% of the HJDs. Under reverse bias, the p-NiO_x_ effectively depleted the channel layer, achieving a leakage current four orders of magnitude lower than that of the SBDs. With a L_AC_ = 12 µm, the BV reached 1.55 kV. Hence, FERs amalgamate the advantages of HJDs and SBDs. Qin et al. [37] deposited p-NiO on the surface of β-Ga_2_O_3_ to form a RESURF structure to deplete the charge in the channel, thereby minimizing surface electric field concentration, as shown in Figure 15f. Charge balance was achieved with a p-NiO thickness of 75 nm, resulting in a BV exceeding 10 kV for a L_AC_ > 30 µm, a R_on_ of 270 mΩ·cm^2^, and a persistent performance above 10 kV even at 200 °C, thus fully demonstrating the potential of β-Ga_2_O_3_ power devices.

Furthermore, the development of lateral flexible SBDs based on β-Ga_2_O_3_ has been explored. Due to the crystal structure of β-Ga_2_O_3_, strain induces microcracks in the nanofilm, leading to device performance degradation, which will not be recounted here in detail [252,253].

### 4.4. Summary

The preceding sections have delineated the performance characteristics of various β-Ga_2_O_3_ power SBD structures. In the simple structure, the high-voltage potential intrinsic to β-Ga_2_O_3_ material was unveiled by eliminating unreliable surface layers. Field plate structures were employed to mitigate electric field concentration at the electrode edges, while edge termination further reduced geometric catastrophe in devices. The trench structure, through the side MOS structure’s RESURF effect, significantly improved the reverse performance of the devices, albeit at the expense of forward performance due to the reduced conduction area. The mesa structure shifted the geometric discontinuities between the electrodes and β-Ga_2_O_3_ inwards into the β-Ga_2_O_3_ material itself, and device performance was further enhanced through the introduction of the MOS structure. Heterojunction diodes exhibited the most exceptional comprehensive performance and have been the subject of the broadest research. The lateral structure achieved the highest BV but has a sizable R_on_. Table 2 comprehensively compares devices with high performance across the various structures.

## 5. Surge Current Ruggedness and Thermal Management

In practical circuit applications, anomalies such as short circuits, overloads, and arcing can induce surge currents. Under such conditions, β-Ga_2_O_3_ SBDs generate substantial heat instantaneously. Given the inherently low thermal conductivity of the β-Ga_2_O_3_ material, this leads to rapid temperature increases, profoundly affecting device performance and even causing thermal breakdown, resulting in irreversible circuit damage. Thus, surge current robustness and thermal management for devices are paramount in real applications.

Experiments have demonstrated that integrating β-Ga_2_O_3_ heterostructures onto high-thermal-conductivity substrates can effectively enhance device heat dissipation. One of the most effective solutions is to utilize diamond substrates to grow Ga_2_O_3_ thin films, due to the excellent thermal conductivity of diamond, which can effectively dissipate heat [254,255,256,257,258]. However, due to lattice mismatch, the quality of β-Ga_2_O_3_ films grown by heteroepitaxy is often poor, limiting device performance [259,260,261]. In 2019, Xu et al. [262] first reported the heterogeneous integration of 2-inch β-Ga_2_O_3_ films onto 4H-SiC and Si (001) substrates using an ion-cutting process. Due to the high thermal conductivity of the substrate, β-Ga_2_O_3_ MOSFETs fabricated on the heterogeneous integration wafers exhibited excellent thermal stability at 500 K. Infrared thermal imaging analysis revealed that, under the same power, the temperature rise of SBDs on the β-Ga_2_O_3_/SiC heteroepitaxial wafer was only a quarter of that on the β-Ga_2_O_3_ wafer, demonstrating that the combination of β-Ga_2_O_3_ thin film with a high-thermal-conductivity SiC substrate effectively promoted the heat dissipation of β-Ga_2_O_3_-based devices [263].

**Figure 16:**
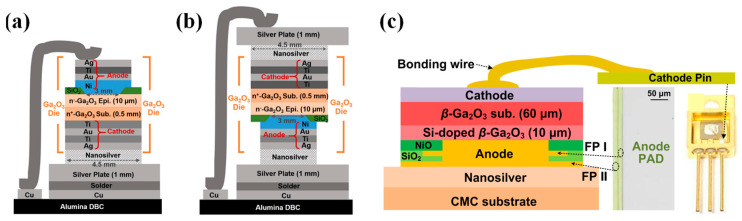
Schematic of β-Ga_2_O_3_ SBDs with (**a**) bottom-side-cooling package and (**b**) double-side-cooling flip-chip package [269]; (**c**) schematic structure of thin-body β-Ga_2_O_3_ SBD with flip-chip packaging [271].

However, for most β-Ga_2_O_3_ SBDs that have vertical structures, this approach is not applicable. Researchers have discovered through simulation and experimentation that substrate thinning and junction-side cooling can effectively lower the thermal resistance of β-Ga_2_O_3_ SBDs, thereby enhancing their surge current robustness [264,265,266,267]. Zhou et al. [268] fabricated a field plate heterojunction diode and reduced the substrate from 650 µm to 150 µm, enabling the device to withstand a surge current of 50 A. Xiao et al. [269] applied bottom cooling and double-side cooling for device packaging for the first time, as shown in Figure 16a,b, achieving peak surge currents of 37.5 A and 68 A, respectively, demonstrating the feasibility of double-side cooling. Due to the small temperature dependence of the R_on_, the thermal runaway is significantly reduced, resulting in a peak surge current to rated current ratio higher than that of commercial SiC SBDs of the same level. Later, by using a transient dual-interface method, they measured junction-case thermal resistances of 1.43 K/W and 0.5 K/W for bottom cooling and junction-side cooling, respectively, with the latter being lower than that of commercial SiC SBDs of the same level, proving the effectiveness of junction-side cooling for β-Ga_2_O_3_ devices [270]. Gong et al. [271] thinned the substrate to 70 µm and employed junction-side cooling packaging, as shown in Figure 16c. Compared to non-thinned devices, the junction-case thermal resistance was reduced from 2.71 K/W to 1.48 K/W, and the surge current resistance improved from 47A to 58A. When the device was applied to a 150 W system-level power conversion circuit, a record-breaking conversion efficiency of 98.9% was achieved, revealing the prospect of device-level thermal management for high-power β-Ga_2_O_3_ SBDs. Despite the low thermal conductivity of the β-Ga_2_O_3_ material, with appropriate thermal management methods, β-Ga_2_O_3_ diodes can achieve temperature rises equivalent to or even lower than those of SiC diodes, effectively enhancing the robustness of their surge current.

## 6. Circuit Application and Reliability

**Figure 17:**
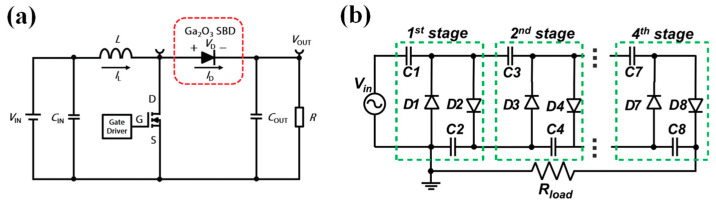
(**a**) Schematic of the DC-DC converter based on the β-Ga_2_O_3_ SBDs [274]; (**b**) circuit of the four-stage HW series CW voltage multiplier circuit with β-Ga_2_O_3_ HJBS diodes [239].

In the past decade, the rapid advancement of β-Ga_2_O_3_ SBDs has led to significant improvements in their performance, prompting researchers to explore their potential in power circuit applications. Oishi et al. [272] fabricated β-Ga_2_O_3_ FP SBDs and demonstrated their application in a single-ended parallel microwave power-rectifying circuit, successfully converting a 1.4 GHz microwave input signal at a power level of 23.7 dBm to a DC output of 43 mV. Guo et al. conducted extensive studies on the application of β-Ga_2_O_3_ SBDs in DC-DC converters with the circuit depicted in Figure 17a, achieving a peak conversion efficiency of 95.81% [155,273,274]. Wu et al. [239] employed β-Ga_2_O_3_ HJBS diodes and commercial SiC SBDs to construct a hybrid half-wave (HW) Cockcroft–Walton (CW) voltage multiplier, as shown in Figure 17b. Compared with a four-stage hybrid voltage multiplier based on SiC SBDs, it exhibited a nearly equivalent multiplication factor of up to 3.81 and a circuit efficiency of approximately 86.07%. These findings underscore the substantial potential for the application of β-Ga_2_O_3_ devices in power circuits.

Power semiconductor devices are often expected to operate under high-load conditions and sustain prolonged durations, thus rendering their long-term operational reliability as an inevitable challenge. Wilhelmi et al. [275] fabricated large-area β-Ga_2_O_3_ SBDs using N ion-implanted terminations and SiO_2_ field plates, achieving a BV greater than 1.1 kV. When deployed in a 400 V to 200 V step-down converter, the devices were able to operate stably for several hours at switching frequencies up to 350 kHz, with an efficiency markedly superior to fast recovery silicon diodes, particularly under high-frequency and high-power conditions. Zhou et al. [207] ascertained the reliability of beveled mesa NiO/β-Ga_2_O_3_ HJDs following more than one million dynamic breakdown events at a peak overvoltage of 1.2 kV, with no significant performance degradation observed. Additionally, the devices demonstrated an up to 98.5% conversion efficiency and a stable operational capability of 100 min in a 500 W power conversion circuit.

In conclusion, current studies on the circuit application and reliability verification of β-Ga_2_O_3_ SBD devices remain sparse, necessitating extensive research to further their practical implementation.

## 7. Summary and Prospect

This article provides a summary of the research progress on β-Ga_2_O_3_ power diode devices. Due to the low cost and high performance of β-Ga_2_O_3_ materials, they have significant potential for surpassing GaN and SiC as one of the leading materials in the high-power electronics market. Presently, research on Ga_2_O_3_ devices is in its infancy, yet the future holds promising prospects and opportunities. At the same time as optimizing materials and structures to enhance the performance of β-Ga_2_O_3_ power devices, the following points will have a considerable impact on the practical applications of β-Ga_2_O_3_ power diode devices.

(a) The development of iridium-free β-Ga_2_O_3_ single-crystal manufacturing technologies and the improvement of crystal quality will greatly facilitate the practical application of β-Ga_2_O_3_.
(b) The mobility of NiO layers prepared by sputtering is significantly lower compared to β-Ga_2_O_3_, which limits the performance of HJDs to some extent. The realization of effective p-type doped Ga_2_O_3_, or developing alternative high-performance p-type materials, will further enhance the performance of β-Ga_2_O_3_ HJDs.
(c) Due to the very low thermal conductivity of β-Ga_2_O_3_ materials, there is still a need for better β-Ga_2_O_3_ thermal management methods and further research on device reliability.

## Figures and Tables

**Table 1:** Comparative analysis of performance parameters of different power semiconductor materials [19,96].

Material Parameters	Si	GaAs	4H-SiC	GaN	β-Ga_2_O_3_	Diamond
Bandgap, E_g_ (eV)	1.1	1.43	3.25	3.4	4.7–4.9	5.5
Electron mobility, µ (cm^2^/V·s)	1480	8400	1000	1250	300	2000
Breakdown field, E_c_ (MV/cm)	0.3	0.4	2.5	3.3	8	10
Saturation velocity, v_s_ (10^7^ cm/s)	1	1.2	2	2.5	1.8–2	1
Dielectric constant, ε	11.8	12.9	9.7	9	10	5.5
Thermal conductivity, λ (W/cm·K)	1.5	0.5	4.9	2.3	0.1–0.3	20
Baliga, εµE_c_^3^	1	14.7	317	846	3444	24,660

**Table 2:** Comparison of electrical performances for different-structure β-Ga_2_O_3_ SBDs.

Device Structure	Anode Area	V_on_ (V)	I_F_	R_on_	BV	PFOM (GW/cm^2^)	References
				(mΩ·cm^2^)	(kV)		
w/o unreliable surface	Φ100 µm	—	—	2.25	1.72	1.32	[138]
BTO FP	Φ50–300 µm	—	—	6.9–8.7	2.1	0.51–0.64	[157]
Mg ion implantation ET	Φ180 µm	—	200 A/cm^2^@2 V	5.1	1.55	0.47	[165]
Oxygen-annealing ET	Φ110 µm	—	—	4.1	1.8	0.78	[172]
SiO_2_ ET	Φ180 µm	1.45	400 A/cm^2^@3 V	3.4	6	10.6	[173]
Trench with FP	100 × 150 µm^2^	—	—	8.8 (pulsed)	2.89	0.95 (pulsed)	[188]
Trench with BTO dielectric	200 × 200 µm^2^	—	—	6.8	>3	>1.32	[190]
	1 × 1 mm^2^	0.9	3.7 A@5 V (pulsed)	7.1	1.8	0.46	
	2 × 2 mm^2^	—	15 A@5 V (pulsed)	10.8	1.4	0.18	
MT with PtO_x_	Φ100 µm	1.45	—	7.33	2.74	1.02	[196]
MT with PBT	Φ100 µm	1.48	—	3.6	1.71	0.8	[199]
AHJD	Φ100 µm	1.72	—	2.5	2.66	2.83	[218]
Beveled-mesa HJD	1 × 1 mm^2^	1.82	20 A@ < 12 V	1.9	1.95	2	[219]
Double-layered NiO HJD	Φ100 µm	1.9–2.1	>100 A/cm^2^@3 V	7.9	8.9	10.2	[226]
	1 × 1 mm^2^	—	4.1 A@10 V	1.8	>4	9	
HJD with SiO_x_/SiO_x_ FP	1 × 1 mm^2^	~2	1 A@3 V	5.4	7	9.2	[228]
HJD with BFP	Φ60 µm	1.6	—	1.12	2.41	5.18	[229]
HJD with FP and ET	Φ150 µm	1.8	~400 A/cm^2^@5 V	5.24	8.32	13.2	[39]
HJD with JTE	Φ100 µm	—	—	3.9	>3.2	2.5–2.7	[244]
Vertical SJ	—	1	—	0.7	2	5.71	[247]
Lateral with dual FP and PAAT	L_AC_=90 µm	1.02	7.43 mA/mm@5 V	485	>10	>0.21	[38]
Lateral SJ with BTO	L_AC_/W_Fin_ = 5 µm/2 µm	—	—	1.65	1.49	1.34	[250]
Lateral with charge balance	L_AC_>30 µm	1	—	270	>10	>0.37	[37]

## Data Availability

Data are contained within the article.
